# Financial toxicity in people with chronic kidney disease undergoing hemodialysis treatment

**DOI:** 10.1590/0034-7167-2022-0671

**Published:** 2023-09-04

**Authors:** Emanuele Cristina de Sousa Silva, Maria de Fátima Mantovani, Luciana de Alcantara Nogueira, Mahara Louise Küchler, Christian Carla Aparecida Volski Cassi, Luciana Puchalski Kalinke

**Affiliations:** I Universidade Federal do Paraná. Curitiba, Paraná, Brazil

**Keywords:** Chronic Kidney Disease, Financial Toxicity, Health Care Costs, Adult Health, kidney Dialysis, Enfermedad Renal Crónica, Toxicidad Financiera, Costos de la Atención en Salud, Salud del Adulto, Diálisis de Riñón, Doença Renal Crônica, Toxicidade Financeira, Custos de Cuidados de Saúde, Saúde do Adulto, Diálise Renal

## Abstract

**Objective:**

to assess the financial toxicity of people with chronic kidney disease undergoing hemodialysis treatment.

**Method:**

a descriptive analytical cross-sectional study, carried out with 214 people, between February and May 2022. For data collection, a sociodemographic and clinical instrument and the COmprehensive Score for financial Toxicity were used. For analysis, the Odds Ratio, ANOVA and Cronbach’s alpha tests were used.

**Results:**

the mean financial toxicity score was 20.30. Women with a monthly family income of at most two minimum wages are more likely to have some degree of financial toxicity (Odds Ratio: 0.85; 0.76).

**Conclusion:**

financial toxicity was identified to different degrees and varied according to sociodemographic and clinical characteristics. Measuring financial toxicity can help nurses plan care and develop strategies to avoid interrupting treatment.

## RESUMO

**Objetivo:**

avaliar a toxicidade financeira de pessoas com doença renal crônica em tratamento hemodialítico.

**Método:**

estudo descritivo analítico com corte transversal, realizado com 214 pessoas, entre os meses de fevereiro e maio de 2022. Para coleta de dados, utilizou-se instrumento sociodemográfico e clínico e o *COmprehensive Score for financial Toxicity*. Para análise, utilizaram-se os testes *Odds Ratio,* ANOVA e alfa de Cronbach.

**Resultados:**

a média do escore da toxicidade financeira foi de 20,30. Pessoas do sexo feminino e com renda familiar mensal de no máximo dois salários mínimos têm maiores chances de apresentar algum grau de toxicidade financeira (*Odds Ratio:* 0,85; 0,76).

**Conclusão:**

a toxicidade financeira foi identificada em diferentes graus e variou de acordo com as características sociodemográficas e clínicas. A mensuração da toxicidade financeira pode auxiliar o enfermeiro no planejamento do cuidado e na elaboração de estratégias para evitar a interrupção do tratamento.

## RESUMEN

**Objetivo:**

evaluar la toxicidad financiera de personas con enfermedad renal crónica en tratamiento de hemodiálisis.

**Método:**

estudio transversal analítico descriptivo, realizado con 214 personas, entre febrero y mayo de 2022. Para la recolección de datos se utilizó un instrumento sociodemográfico, clínico y el COmprehensive Score for financial Toxicity. Para el análisis se utilizaron las pruebas de Odds Ratio, ANOVA y alfa de Cronbach.

**Resultados:**

la puntuación media de toxicidad financiera fue de 20,30. Las mujeres con un ingreso familiar mensual de a lo sumo dos salarios mínimos tienen más probabilidades de tener algún grado de toxicidad financiera (Odds Ratio: 0,85; 0,76).

**Conclusión:**

la toxicidad financiera se identificó en diferentes grados y varió según las características sociodemográficas y clínicas. Medir la toxicidad financiera puede ayudar a las enfermeras a planificar la atención y desarrollar estrategias para evitar la interrupción del tratamiento.

## INTRODUCTION

Illness has emotional and financial consequences for patients and sometimes for family members^(1)^. Chronic kidney disease (CKD) is considered a public health concern, as it has a high incidence, generates high rates of morbidity and mortality, has a prolonged, progressive and insidious course, in addition to being often silent, showing no signs or symptoms for most of its evolution^(2)^.

People with CKD who need to undergo hemodialysis (HD) have their routine changed and often cannot stay in the job market, which increases their financial difficulties. This is because, depending on their clinical condition, there is a need to undergo HD three times a week, which makes it difficult to reconcile treatment and work. Allied to decreased income, there may be an increase in expenses, due to food and transportation to carry out treatment, which can cause some degree of financial toxicity (FT) in people with CKD.

FT is defined as the harmful impact experienced by patients who cannot afford to pay for treatment and bear the extra expenses inherent to their condition. The expression includes health cost burden and issues not related to treatment, but that can influence it financially, becoming a barrier to the necessary medical care^(3)^.

According to the international literature, there are several consequences caused by FT, such as difficulty in maintaining care with treatment and basic needs, such as food and housing, increased cases of anxiety, depression and sleep disorders, worsening of clinical picture, increase in the number of hospitalizations, among others^(4-5)^.

FT is measured using the COmprehensive Score for financial Toxicity (COST) instrument from the Functional Assessment of Chronic Illness Therapy (FACIT) group. The COST was validated in Brazil^(6)^ in a survey conducted with patients with cancer. Another study^(7)^ validated the COST among patients with chronic disease and demonstrated that the instrument has the potential to be used in populations other than those with cancer to assess FT in diseases such as CKD.

There are the peculiarities of funding related to CKD and HD treatment in Brazil as well as changes in the lives of people and their families, with a significant number of people undergoing HD in the country, around 144,779^(8)^. Moreover, the COST, despite being an instrument not yet used in CKD, can help nurses and other health team members to effectively develop strategies to minimize financial impacts. Therefore, the question is: is there an impact of FT on people undergoing HD treatment?

## OBJECTIVE

To assess the FT of people with CKD undergoing HD treatment.

## METHOD

### Ethical aspects

This study was assessed and approved by the Research Ethics Committee of the *Universidade Federal do Paraná*. Data collection instruments were authorized for use by developers and translators. The Informed Consent Form was obtained from all individuals involved in the study in writing.

### Study design, location, period and questionnaires used

This is a descriptive analytical cross-sectional study, which followed the STrengthening the Reporting of OBservational studies in Epidemiology (STROBE) recommendations. It is part of a thematic project entitled “Financial toxicity in chronic illness” and was extracted from an academic master’s course completion work.

It was carried out with 214 people diagnosed with CKD who were undergoing HD treatment in a service that performs HD, peritoneal dialysis, kidney transplantation and tests in people from the public system, with health insurance and paid consultation, consisting of four specialized units, located in Curitiba and region metropolitan.

Data collection was carried out from February to May 2022, by consulting medical records and applying two questionnaires: 1) sociodemographic and clinical, with questions related to age, sex, race, marital status, education, financial situation, family and health, time since CKD diagnosis and HD, medication use, alcohol consumption and smoking; 2) COST, which has questions about patients’ financial concerns, frustration for not contributing to the income, satisfaction with the current financial situation, among others. The COST has 12 items, and responses are arranged on a five-point Likert-type scale, ranging from zero (not at all) to four (very much). Item 12 is a summary item and has no scoring value, and questions 2, 3, 4, 5 8, 9 10 are reversed. The score ranges from 0 to 44, as the higher the score, the greater the financial well-being and the lower the FT.

### Sample, and inclusion and exclusion criteria

People with CKD, aged equal to or greater than 18 years, who started HD treatment six months ago or more were included. People with CKD on HD who have communication difficulties and/or mental limitations, recorded in their medical record, were excluded.

**Figure 1:**
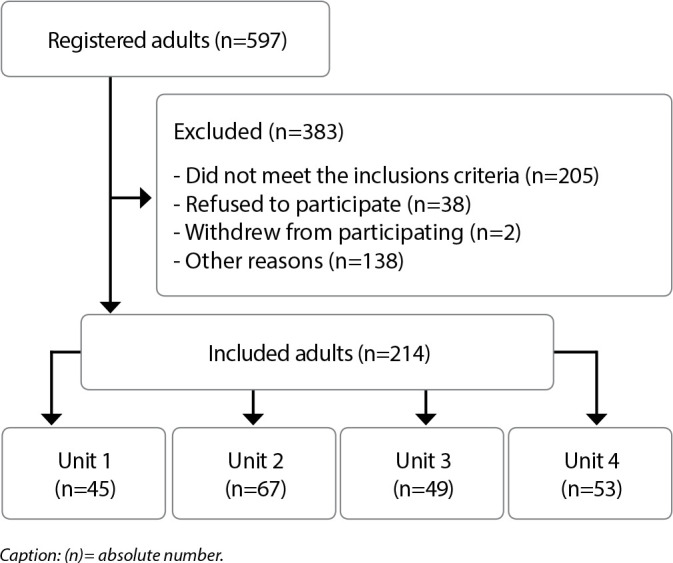
Recruitment flowchart of people with chronic kidney disease undergoing hemodialysis to participate in the study, Curitiba, Paraná, Brazil, 2022

Sampling was stratified and calculated based on the attendances at each of the clinics (Figure 1) for the year 2021. Participation was voluntary and recruitment was for convenience, with all those eligible being invited, according to data assessment in medical records, which were on site at the time of data collection.

### Analysis of results, and statistics

For sociodemographic and FT data analysis, the classification made by a Japanese study^(9)^ was used, in which the FT degree is considered based on the score. According to the study authors, degree 0 are those patients whose score is greater than 26, which means no impact; degree 1, score between 14-25, corresponding to mild impact; degree 2, score ranging from 1 to 13, representing moderate impact; degree 3, score 0, with high impact.

Descriptive statistics techniques were used to identify sociodemographic characteristics by FT degree. All tests were performed with 5% significance. Crude estimates of Odds Ratio (OR) were obtained for the association between the variables of interest as well as adjusted Odds Ratio (adjusted OR) for the complete model and their respective 95% Confidence Intervals (95%CI) and Bayesian 95% Confidence Interval (redCI). A one-way ANOVA analysis was used to determine FT variability among participating clinics, and COST Cronbach’s alpha was calculated for use in CKD.

## RESULTS

Of the 214 people with CKD undergoing HD treatment, 54.67% (n=117) were male; 52.80% (n=113) were aged 60 years or older; 53.52% (n=114) were married or in a consensual union; 54.67% (n=117) had less than nine years of schooling. As for monthly family income, 24.0% (n=51) of the sample declared an income between 1 and 2 minimum wages.

With regard to clinical characteristics, 78.97% (n=169) reported receiving care from the Unified Health System (SUS - *Sistema Único de Saúde*). Regarding living with other comorbidities in addition to CKD, 81.7% (n=175) had some chronic disease; 12.2% (n=26) reported having a family history of CKD; 59.3%(n=128) declared HD for 1-5 years; and 88.2% (n=188) used continuous medication.

With regard to life habits, 69.69% (n=147) of participants were non-smokers and 27.10% were former smokers. With regard to alcohol consumption, 72.89% (n=156) declared that they did not consume alcohol and 22.89% (n=49) consumed alcoholic beverages, having suspended use after receiving CKD diagnosis.

Regarding FT, the mean of the total score, considering all data collection units, was 20.30. Table 1 shows the mean FT score in each of the units and the total mean and evidences a higher FT in unit 1, with a mean of 16.82.

**Table 1:** Mean financial toxicity score per clinical unit, Curitiba, Paraná, Brazil, 2022

	Cost		
	**Mean**	**SD**	*p* value^*^
UNIT			0.0256
Unit 1	16.82	8.10	
Unit 2	20.98	8.06	
Unit 3	21.34	9.81	
Unit 4	21.42	8.31	
Total	20.30	8.69	

*Caption: SD - standard deviation.*

* *P value extracted from the analysis of variance (ANOVA) table.*

Table 2 shows the correlation between sociodemographic data and FT based on the Japanese classification^(9)^. It was evidenced that, of all participants (n=214), 71% had some degree of FT, being 102 (48%) mild FT degree, 50 (23%), moderate degree, one (0.46%), high degree, and only 61 (29%) had no FT degree.

**Table 2:** Descriptive analysis between sociodemographic variables and financial toxicity degrees, Curitiba, Paraná, Brazil, 2022

	Financial Toxicity Degrees							
	No degree		Mild degree		Moderate degree		High degree	
	(n=61)	95%CI	(n=102)	95%CI	(n=50)	95%CI	(n=1)	95%CI
Sex								
Female	20	32.8% (22.3-45.3)	47	46.1% (36.7-55.7)	30	60.0% (46.2-72.4)	0	0.0% (0.0-79.3)
Male	41	67.2% (54.7-77.7)	55	53.9% (44.3-63.3)	20	40.0% (27.6-53.8)	1	100.0% (20.7-100.0)
Age								
18 to 60	19	31.1% (20.9-43.6)	49	48.0% (38.6-57.6)	32	64.0% (50.1-75.9)	1	100.0% (20.7-100.0)
60 and over	42	68.9% (56.4-79.1)	53	52.0% (42.4-61.4)	18	36.0% (24.1-49.9)	0	0.0% (0.0-79.3)
Marital status								
Single	8	13.1% (6.9-24.2)	15	14.7% (9.1-22.9)	13	26.0% (15.9-39.6)	0	0.0% (0.0-79.3)
Married or common-law marriage	37	60.7% (49.0-72.9)	55	53.9% (44.3-63.3)	21	42.0% (29.4-55.8)	1	100.0% (20.7-100.0)
Separated/divorced/widowed	15	24.6% (15.8-37.2)	31	30.4% (22.3-39.9)	16	32.0% (20.8-45.8)	0	0.0% (0.0-79.3)
Not reported	1	1.0% (0.0-6.0)	1	1.0% (0.2-5.3)	0	0.0% (0.0-7.1)	0	0.0% (0.0-79.3)
Education								
< 9	30	49.2% (37.1-61.4)	63	61.8% (52.1-70.6)	23	46.0% (33.0-59.6)	1	100.0% (20.7-100.0)
9 to 12	14	23.0% (14.2-34.9)	20	19.6% (13.1-28.4)	15	30.0% (19.1-43.8)	0	0.0% (0.0-79.3)
13 to 16	12	19.7% (11.6-31.3)	11	10.8% (6.1-18.3)	3	6.0% (2.1-16.2)	0	0.0% (0.0-79.3)
>16	2	3.3% (0.9-11.2)	4	3.9% (1.5-9.7)	3	6.0% (2.1-16.2)	0	0.0% (0.0-79.3)
Not reported	3	4.9% (1.7-13.5)	4	3.9% (1.5-9.7)	6	12.0% (5.6-23.8)	0	0.0% (0.0-79.3)
Type of cost of treatment								
Private	12	19.7% (11.6-31.3)	19	18.6% (12.3-27.3)	14	28.0% (17.5-41.7)	0	0.0% (0.0-79.3)
SUS	49	80.3% (68.7-88.4)	83	81.4% (72.7-87.7)	36	72.0% (58.3-82.5)	1	100.0% (20.7-100.0)
Family income monthly								
Less than 1 MW	0	0.0% (0.0-5.9)	12	11.8% (6.9-19.4)	14	28.0% (17.5-41.7)	0	0.0% (0.0-79.3)
1 to 2 MW	7	11.5% (5.7-21.8)	28	27.5% (19.7-36.8)	15	30.0% (19.1-43.8)	1	100.0% (20.7-100.0)
2 to 3 MW	17	27.9% (18.2-40.2)	22	21.6% (14.7-30.5)	9	18.0% (9.8-30.8)	0	0.0% (0.0-79.3)
3 to 4 MW	6	9.8% (4.6-19.8)	10	9.8% (5.4-17.1)	1	2.0% (0.4-10.5)	0	0.0% (0.0-79.3)
4 to 5 MW	3	4.9% (1.7-13.5)	2	2.0% (0.5-6.9)	2	4.0% (1.1-13.5)	0	0.0% (0.0-79.3)
5 MW or more	13	21.3% (12.9-33.1)	5	4.9% (2.1-11.0)	1	2.0% (0.4-10.5)	0	0.0% (0.0-79.3)
Not reported	15	24.6% (15.5-36.7)	23	22.5% (15.5-31.6)	8	16.0% (8.3-28.5)	0	0.0% (0.0-79.3)

*Subtitle: (n) - absolute number; MW - minimum wage; CI - confidence interval; SUS - Unified Health System.*

*Note:*

* It was considered the Brazilian minimum wage in force in the year 2022 of R$ 1.212.00 (one thousand, two hundred and twelve reais), or US$242.20.

It was possible to verify that, among the study participants who suffer a mild FT degree (n=102), 55 (53.9%) are male. When observing the results of those with a moderate degree (n=50), 30 (60.0%) correspond to females, indicating that, in this sample, women have a higher FT degree.

With regard to age group, among those classified as moderate (n=50), 32 (64.0%) were between 18 and 60 years old. As for monthly family income, it was possible to observe that all with family income below the minimum wage (n=26) had some degree of FT; of these, 12 (11.8%) and 14 (28.0%) had a mild and moderate degree (Table 2).

Regarding the type of cost of treatment, it was possible to verify that, of the 45 participants who do it privately, 19 (18.6%) and 14 (28.0%) had a mild and moderate FT degree.

Table 3 shows the relationship between participant clinical characteristics and FT, revealing that 175 (81.7%) have other comorbidities in addition to CKD and, of these, 126 (72.0%) had some degree of FT.

**Table 3:** Descriptive analysis between clinical characteristics, lifestyle habits and financial toxicity degrees. Curitiba, PR, Brazil, 2022

	Financial Toxicity Degrees							
	No degree		Mild degree		Moderate degree		High degree	
	(n=61)	95%CI	(n=102)	95%CI	(n=50)	95%CI	(n=1)	95%CI
Comorbidities accompanying CKD								
Yes	49	80.3% (68.7-88.4)	84	82.4% (73.8-88.5)	41	82.0% (69.2-90.2)	1	100.0% (20.7-100.0)
No	11	18.0% (10.4-29.5)	18	17.6% (11.5-26.2)	9	18.0% (9.8-30.8)	0	0.0% (0.0-79.3)
Not reported	1	1.6% (0.3-8.7)	0	0.0% (0.0-3.6)	0	0.0% (0.0-7.1)	0	0.0% (0.0-79.3)
CKD family history								
No	56	91.8% (82.2-96.4)	87	85.3% (77.1-90.9)	42	84.0% (71.5-91.7)	1	100.0% (20.7-100.0)
Yes	5	8.2% (3.6-17.8)	14	13.7% (8.4-21.7)	7	14.0% (7.0-26.2)	0	0.0% (0.0-79.3)
Not reported	0	0.0% (0.0-5.9)	1	1.0% (0.2-5.3)	1	2.0% (0.4-10.5)	0	0.0% (0.0-79.3)
Time since CKD diagnosis (years)								
< 1	8	13.1% (6.8-23.8)	9	8.8% (4.7-15.9)	6	12.0% (5.6-23.8)	0	0.0% (0.0-79.3)
1 to 5	30	49.2% (37.1-61.4)	50	49.0% (39.5-58.6)	22	44.0% (31.2-57.7)	0	0.0% (0.0-79.3)
6 to 10	11	18.0% (10.4-29.5)	20	19.6% (13.1-28.4)	13	26.0% (15.9-39.6)	1	100.0% (20.7-100.0)
>10	12	19.7% (11.6-31.3)	23	22.5% (15.5-31.6)	9	18.0% (9.8-30.8)	0	0.0% (0.0-79.3)
Hemodialysis duration (years)								
< 1	13	21.3% (12.9-33.1)	15	14.7% (9.1-22.9)	9	18.0% (9.8-30.8)	0	0.0% (0.0-79.3)
1 to 5	37	60.7% (48.1-71.9)	62	60.8% (51.1-69.7)	27	54.0% (40.4-67.0)	1	100.0% (20.7-100.0)
6 to 10	5	8.2% (3.6-17.8)	9	8.8% (4.7-15.9)	8	16.0% (8.3-28.5)	0	0.0% (0.0-79.3)
> 10	6	9.8% (4.6-19.8)	14	13.7% (8.4-21.7)	6	12.0% (5.6-23.8)	0	0.0% (0.0-79.3)
Not reported	0	0.0% (0.0-5.9)	2	2.0% (0.5-6.9)	0	0.0% (0.0-7.1)	0	0.0% (0.0-79.3)
Number of weekly hemodialysis								
2 times	3	4.9% (1.7-13.5)	0	0.0% (0.0-3.6)	0	0.0% (0.0-7.1)	0	0.0% (0.0-79.3)
3 times	55	90.2% (80.2-95.4)	99	97.1% (91.7-99.0)	48	96.0% (86.5-98.9)	1	100.0% (20.7-100.0)
more than 3 times	3	4.9% (1.7-13.5)	3	2.9% (1.0-8.3)	2	4.0% (1.1-13.5)	0	0.0% (0.0-79.3)
Continuous medication use								
Yes	55	90.2% (80.2-95.4)	88	86.3% (78.3-91.6)	44	88.0% (76.2-94.4)	1	100.0% (20.7-100.0)
No	5	8.2% (3.6-17.8)	14	13.7% (8.4-21.7)	6	12.0% (5.6-23.8)	0	0.0% (0.0-79.3)
Not reported	1	1.6% (0.3-8.7)	0	0.0% (0.0-3.6)	0	0.0% (0.0-7.1)	0	0.0% (0.0-79.3)

*Subtitle: (n) - absolute number; MW - minimum wage; CI - confidence interval; CKD - chronic kidney disease.*

As for CKD diagnosis and HD treatment duration, most had a mild or moderate impact (n=152), and the only participant who had a high impact of FT has 6 to 10 years of CKD diagnosis. With regard to HD duration from one to five years and FT degree, participants had a mild degree, 37 (29.1%), moderate, 62 (48.8%), and one participant had a high FT degree, with this being the HD duration that had the most changes in FT. Of the participants who used continuous medication, most (n=132) had a mild or moderate degree and one had a high FT degree (Table 3).

According to data in Table 4, it was possible to observe that there was statistical significance between FT and some variables, such as sex and income. It is verified that women are more likely to present some degree of FT than men (OR=0.85) as well as people who receive up to 2 minimum wages (OR=0.76).

**Table 4:** Correlation of financial toxicity with gender, family income and medication use, Odds Ratio and respective 95% confidence intervals, Curitiba, Paraná, Brazil, 2022

Variables	OR	95%redCI	adjusted OR	95%redCI
Intercept			2.60	1.99; 3.37
Sex				
Female	1.00	Ref.	1.00	Ref.
Male	0.81	0.70; 0.93	0.85	0.74; 0.98
Age				
18 to 60	1.00	Ref.	1.00	Ref.
60 and above	0.85	0.74; 0.98	0.89	0.76; 1.03
Marital status				
Single	1.00	Ref.	1.00	Ref.
Married or common-law marriage	0.86	0.70; 1.05	0.93	0.71; 1.20
Separated/divorced/widowed	0.98	0.78; 1.22	0.94	0.73; 1.23
Number of children				
0	1.00	Ref.	1.00	Ref.
1 to 3	1.08	0.88; 1.34	1.14	0.87; 1.50
more than 3	1.13	0.89; 1.43	1.13	0.84; 1.55
Education				
< 9	1.00	Ref.	1.00	Ref.
9 to 12	0.98	0.83; 1.17	0.97	0.82; 1.16
13 or more	0.85	0.70; 1.03	0.95	0.78; 1.17
Cost of treatment				
SUS	1.00	Ref.	1.00	Ref.
Private	1.01	0.83; 1.23	1.01	0.83; 1.21
Number of people living in the residence				
1	1.00	Ref.	1.00	Ref.
2	0.82	0.66; 1.01	0.97	0.77; 1.21
3	0.99	0.79; 1.24	1.16	0.92; 1.48
4 or more	0.98	0.79; 1.21	1.05	0.82; 1.35
Monthly family income				
Less than 2 MW	1.00	Ref.	1.00	Ref.
2 or more	0.73	0.64; 0.83	0.76	0.65; 0.88

*Subtitle: MW - minimum wage; redCI - 95% Bayesian confidence interval; OR - Odds Ratio.*

*Note:*

* The Brazilian minimum wage in force in 2022 was considered to be R$ 1.212.00 (one thousand, two hundred and twelve reais), or US$242.20.

When checking item reliability individually in the COST questionnaire, it was possible to observe that the minimum and maximum Cronbach’s alpha coefficient found was 0.81 and 0.84, respectively. Cronbach’s alpha coefficient, considering all items, is 0.84.

## DISCUSSION

This study assessed the FT of people with CKD under HD and correlated the mean score with sociodemographic and clinical data. With regard to sociodemographic data, it was possible to observe the similarity of the results found with two studies. The first is a Brazilian study carried out in Santa Catarina, with 70 people with CKD on HD, in which there was a predominance of males (52.8%), aged between 20 and 86 years, with 41.42% of them were between 40 and 59 years old; 39.99% were over 60 years old; 52.85% were married; and most of them had low levels of education and were retired^(10)^. The second study was conducted in Bahia, with 110 people in a dialysis center, in which the highest percentage was of people aged over 60 years (30.5%) and married (46.7%)^(11)^.

With regard to family income in this study, a quarter of the participants had an income between one and two minimum wages, a value that can show FT, even though care is provided by the public health system. Another study carried out in Brazil with people with cancer identified that the lower the purchasing power, the higher the FT^(6)^.

Although the concept of FT is not recent, in Brazil, the first publication in a scientific journal dates from 2021, revealing how innovative the theme is in the country, highlighting the lack of studies that allow comparisons in different realities and with the public health system^(12)^.

When comparing the FT score of different health systems and diseases, similarity of values is also observed, as in the study carried out in Canada^(13)^, where there is a public health system, which obtained a mean score of 21 with participants with advanced lung cancer, and the one conducted in Brazil^(6)^, with people with cancer undergoing chemotherapy, whose mean score was 18.95 as well as in the studies conducted in the United States of America (USA), with the mean of the score in multiple sclerosis of 17.4^(14)^, in Crohn’s disease^(15)^, 22.0, and in cardiac amyloidosis^(16)^, 25.0.

FT degree in this study may be related to the fact that 78.9% of the sample received treatment through SUS. In the study carried out with people with cancer in Brazil^(6)^, it was evidenced that participants assisted by health insurance and/or who paid for treatment privately had higher mean COST scores, indicating greater financial well-being than those assisted by SUS.

An Australian study, which assessed FT in people with CKD, showed that: 78% reported financial difficulties in the last twelve months; almost half of participants used their savings to cover expenses; 54% of participants were in great difficulty due to the extra expenses of treatment, which represented more than 10% of the family income. Problems were reported to pay for medical care (26%), dental care (25%), medication (23%) as well as difficulty with transportation for treatment (22%) and for buying medication (21%)^(17)^.

In this study, it was possible to observe the lowest degree of FT in people aged over 60 years, corroborating the findings of a multicenter research carried out in Israel and the USA with women with gynecological cancer^(18)^, in which people over 50 years were less prone to incur FT compared to adults in younger age groups. Those with less education had a higher FT degree, similar to the result of research conducted in Malaysia with cancer survivors, in which people with at least high school had better financial well-being, compared to those who had elementary school or no formal education^(19)^, and in the female population with cancer, in which younger age, lower education, no employment relationship and lower income were associated with worse FT^(18)^.

As for the presence of comorbidities in addition to CKD, in this study, 82% of participants with moderate impact of FT had other associated comorbidities, which may increase financial difficulties. This result is similar to those found in other studies, in which 54.7% had a pathology associated with CKD, such as hypertension and Diabetes Mellitus (DM)^(20)^, and in another, in which 76.9% of participants had hypertension^(21)^.

In a study carried out between 2013 and 2017 in the USA, it was observed, when researching 9,000 individuals with DM, that 41.1% of participants faced financial difficulties due to medical expenses and 15.6% were unable to pay their bills. This situation certainly worsens in the presence of two or more chronic diseases that add care with medication, tests, consultations, among others, aggravating FT^(22)^.

With regard to lifestyle, among participants in this study with moderate and high FT levels, more than half used continuous medication. Similar data was found in a study^(6)^ with people with cancer, which found that, of the total number of participants, 60.32% used frequent medication.

Regarding the correlation between FT and sociodemographic data, in this study, female participants with income of up to two minimum wages were more likely to have some degree of FT than the other participants. Similar data were obtained in a study with people with DM, in which women with health care paid for by the government, aged between 45 and 59 years, without employment and with low education, had a worse COST score (P <0.01)^(23)^. Income of up to one minimum wage was also a factor for worsening FT in a study in people undergoing chemotherapy treatment^(6)^.

With regard to COST reliability for use in a population with chronic disease, this study obtained an excellent result^(24)^, similar to that found in a North American study, which obtained a Cronbach’s alpha of 0.84, demonstrating that COST can be used in populations with chronic disease.

### Study limitations

The limitations of this study are due to type of sampling for convenience, a fact that makes it impossible to generalize the results, although it reveals important findings related to FT in CKD.

### Contributions to nursing, health, or public policies

It is believed that this study brings contributions to practice, as it highlights the presence of FT among people with CKD on HD, as these data may guide the development of more effective interventions to minimize the side effects that the disease and treatment can generate.

## CONCLUSION

The result of the COST score of the studied sample indicates the existence of FT in different degrees, and most participants presented a mild degree. Those with lower income and females are more likely to have some level of FT related to CKD and HD treatment.

This is the first study to apply the COST instrument, recently translated into Brazilian Portuguese, to the population with CKD undergoing HD treatment. The results suggest that this population has FT degrees that need to be explored in research that compares the impact of CKD in different services and with different forms of costing treatment.
